# Structural and Functional Studies of Influenza Virus A/H6 Hemagglutinin

**DOI:** 10.1371/journal.pone.0134576

**Published:** 2015-07-30

**Authors:** Fengyun Ni, Elena Kondrashkina, Qinghua Wang

**Affiliations:** 1 Verna and Marrs McLean Department of Biochemistry and Molecular Biology, Baylor College of Medicine, Houston, Texas, United States of America; 2 Life Sciences Collaborative Access Team (LS-CAT), Synchrotron Research Center, Northwestern University, Argonne, Illinois, United States of America; University of Rochester Medical Center, UNITED STATES

## Abstract

In June 2013, the first human infection by avian influenza A(H6N1) virus was reported in Taiwan. This incident raised the concern for possible human epidemics and pandemics from H6 viruses. In this study, we performed structural and functional investigation on the hemagglutinin (HA) proteins of the human-infecting A/Taiwan/2/2013(H6N1) (TW H6) virus and an avian A/chicken/Guangdong/S1311/2010(H6N6) (GD H6) virus that transmitted efficiently in guinea pigs. Our results revealed that in the presence of HA_1_ Q226, the triad of HA_1_ S137, E190 and G228 in GD H6 HA allows the binding to both avian- and human-like receptors with a slight preference for avian receptors. Its conservation among the majority of H6 HAs provides an explanation for the broader host range of this subtype. Furthermore, the triad of N137, V190 and S228 in TW H6 HA may alleviate the requirement for a hydrophobic residue at HA_1_ 226 of H2 and H3 HAs when binding to human-like receptors. Consequently, TW H6 HA has a slight preference for human receptors, thus may represent an intermediate towards a complete human adaptation. Importantly, the triad observed in TW H6 HA is detected in 74% H6 viruses isolated from Taiwan in the past 14 years, suggesting an elevated threat of H6 viruses from this region to human health. The novel roles of the triad at HA_1_ 137, 190 and 228 of H6 HA in binding to receptors revealed here may also be used by other HA subtypes to achieve human adaptation, which needs to be further tested in laboratory and closely monitored in field surveillance.

## Introduction

Recent years have witnessed an increasing number of human infections by avian influenza viruses including A(H7N9) [1,2] and A(H10N8) [3] in 2013. In particular, in June 2013, the first human infection by influenza A(H6N1) virus was reported [4–6]. Although the patient eventually recovered from the infection, this incident raised the concern for possible human epidemics and pandemics from avian H6 viruses. The threat is especially immense given that H6 viruses are the most commonly observed subtype in wild aquatic birds [7,8], they infect a broad range of host including mice, ferrets, pigs and humans [9–12], over 30% of the currently circulating H6 viruses recognized human receptors and some even transmitted efficiently in guinea pigs [13], a chimeric 1918 pandemic influenza virus expressing a contemporary H6 hemagglutinin (HA) caused enhanced disease in mammals [14], and inoculation of avian H6N1 virus into dogs led to fever and detectable viruses in the lung [15].

Receptor-binding specificity of HA is a key determinant for viral host range [16–18]. HA molecules of influenza virus isolated from various hosts differ in their ability to recognize receptors in which the linkage between sialic acid (Neu5Ac) and galactose (Gal) of the carbohydrate chain is either α(2,3) or α(2,6). HA molecules of human viruses preferentially bind to α(2,6)-linked receptors, those of avian viruses to α(2,3)-linked receptors, while those of swine viruses to both [19–28]. Thus, α(2,3)- and α(2,6)-linked receptors are also referred to as avian and human receptors, respectively. In order to support efficient airborne transmission among humans to cause influenza epidemics and pandemics, an avian virus has to develop the ability to bind to human-like α(2,6)-linked sialic acid receptors with a concomitant loss of affinity for avian-like α(2,3)-linked sialic acid receptors. Therefore, one critical question in the filed is to understand what sequence and structural changes in HA will endorse an affinity switch from avian receptors to human receptors.

Different subtypes of HA proteins seem to have different sequence requirements for avian-to-human receptor switch [17]. For H2 and H3 HA proteins, the predominant determinants are residues HA_1_ 226 and 228 (L226 and S228 for human HA and Q226 and G228 for avian HA) [24,25,29–33]. However, for H1 HA, residues at HA_1_ 190 and 225 determine receptor specificity: H1 HA with E190/G225, E190/D225 or D190/G225 binds to both avian and human receptors, while H1 HA with D190/D225 or D190/E225 prefers human receptors [17,27,34–36]. For H7 HA, the mutations Q226L and G228S, separately or combined, enhance the binding to human receptors, but do not result in a preference switch from avian to human receptors [37–40].

In this study, we carried out structural and functional investigation on two H6 HA proteins to evaluate their ability, and to understand their molecular mechanism, to bind to avian- or human-like receptors. We found that the HA protein from the human-infecting A/Taiwan/2/2013(H6N1) virus, TW H6 HA, already possesses slight preference for human receptors, thus may act as an intermediate towards a complete human adaptation. Furthermore, TW H6 HA appears to use a novel combination of HA_1_ N137, V190 and S228 to overcome the requirement for a hydrophobic residue at HA_1_ 226 of H2 and H3 HAs when binding to human-like receptors. This triad was identified in 74% of HA sequences from the H6 viruses isolated from Taiwan in the past 14 years, suggesting an elevated threat of this subgroup of H6 viruses to human health.

## Results

### Sequence analysis of H6 HAs between 2000~2014

H6 HA is the most commonly observed subtype in wild aquatic birds [7,8]. We analyzed 737 non-redundant H6 HA sequences between 2000~2014. We found that they are separated into two major phylogenetic groups: Group I (268 sequences) that is mainly composed of H6N2 and H6N6 viruses from Asia, and Group II (459 sequences) which mostly consists of H6N1 and H6N2 viruses from Asia and around the world (S1 **Fig**). In addition, 10 sequences from mallard during 2000~2002 form a distinct group (Early Group, **S1 Fig, Table 1**). Extensive sequence variations were found in the globular head domain surrounding the receptor-binding site formed by the 130-loop, 190-helix, and 220-loop, particularly among Group II H6 HAs (**Table 1**). This observation is consistent with previous reports that the regions surrounding the receptor-binding site are hot spots for antigenic variations [41].

**Table 1:** Amino-acid composition in the receptor-binding site of H6 HA proteins.

HA_1_ Residues	Early Group (10 sequences)	Group I (268 sequences)	Group II (459 sequences)
133	90% S, 10% R	81% S, 19% R	89% S, 7% R, 2% N, 1% K, 1% G
137	100% S	99% S, 0.7% R, 0.3% N	42% K, 36% R, 11% S, 7% N, 3% A, 1% Q
190	100% E	100% E	91% E, 6% V, 1% A, 1% L
228	100% G	99% G, 1% S	94% G, 6% S

### A/chicken/Guangdong/S1311/2010(H6N6) and A/Taiwan/2/2013(H6N1) HAs

Two H6 HA proteins, one from each group, were chosen for structural and functional investigation: A/chicken/Guangdong/S1311/2010(H6N6) (GD H6) in Group I and A/Taiwan/2/2013(H6N1) (TW H6) in Group II. The GD H6 virus, although of chicken origin, was able to infect guinea pigs through direct inoculation or contact [13]. Furthermore, the TW H6 virus caused the first case of H6-mediated human infection [4–6].

Using recombinant proteins purified from insect cells and Bio-Layer Interferometry (BLI)-based OCTET RED96 system (Pall ForteBio), we measured the binding affinity of these H6 HA proteins with avian-like α(2,3)-receptors (3’SLNLN) and human-like α(2,6)-receptors (6’SLNLN) where LN represents lactosamine (Galβ1-4GlcNAc), 3’SLN and 6’SLN represent Neu5Acα(2,3) and Neu5Acα(2,6) linked to LN, respectively. GD H6 HA has an apparent equilibrium dissociation constant of 0.097 μM for avian-like 3’SLNLN receptor, and 5.0 μM for human-like 6’SLNLN receptor (**Table 2**). The overall ~50-fold lower binding affinity of GD H6 HA with 6’SLNLN receptor, compared to 3’SLNLN receptor, seems mainly the result of an about 20-fold decrease in the association rate (**Table 2, S2A Fig**). In sharp contrast, TW H6 HA has similar equilibrium dissociation constants for both types of receptors, 0.4 μM for 3’SLNLN and 0.27 μM for 6’SLNLN (**Table 2**). Comparing to GD H6 HA with 3’SLNLN, TW H6 HA has ~1,000-fold slower association rate and dissociation rate for both 3’SLNLN and 6’SLNLN, thus their overall affinities are comparable (**Table 2, S2B Fig**).

**Table 2:** Binding kinetics of H6 HAs with avian and human receptor analogues.

HA	Glycan	*K*_D_ (M)^a^	*k*_on_ (1/Ms)^a^	*k*_off_ (1/s)^a^
GD H6 HA	3’SLNLN	9.7±0.8 ×10^−8^	1.44±0.11 ×10^6^	1.40±0.04 ×10^−1^
	6’SLNLN	5.0±0.2 ×10^−6^	7.5±0.3 ×10^4^	3.7±0.6 ×10^−1^
TW H6 HA	3’SLNLN	4.0±0.1 ×10^−7^	1.32±0.02 ×10^3^	5.3±0.1 ×10^−4^
	6’SLNLN	2.70±0.05 ×10^−7^	2.38±0.03 ×10^3^	6.42±0.08 ×10^−4^

^a^ *K*_D_: Apparent equilibrium dissociation constant calculated as *k*_off_/*k*_on_; *k*_on_: association rate from the association curves; *k*_off_: dissociation rate from the dissociation curves.

### Structures of GD and TW H6 HA proteins

**Fig 1:**
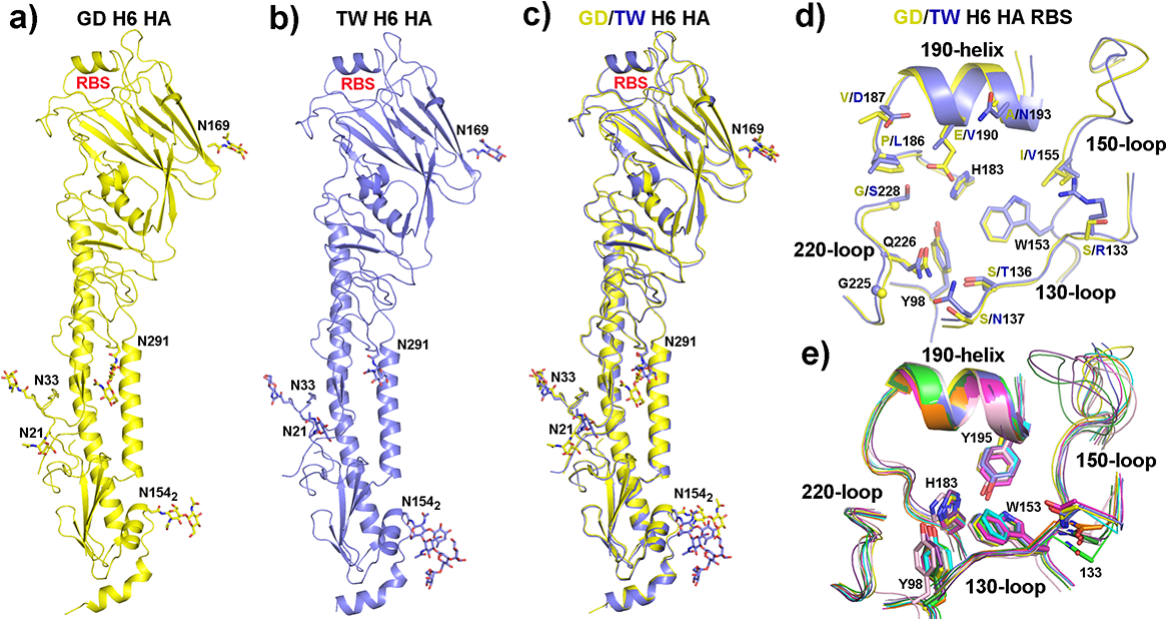
The overall structures of GD and TW H6 HAs. **a).** One monomer of GD H6 HA (in yellow color) with the glycans (in stick model). The receptor-binding site (RBS) is labeled. **b).** One monomer of TW H6 HA (in blue color) with the glycans (in stick model). **c).** Superposition of the monomers of GD (in yellow color) and TW (in blue color) H6 HAs. **d).** Comparison of the receptor-binding sites of GD (in yellow) and TW (in blue) H6 HAs. Highlighted are the residues at or surrounding the receptor-binding site. All residues are in H3 HA numbering. **e).** Comparison of the receptor-binding sites of GD (in yellow) and TW (in blue) H6 HAs with H1 HA from A/Brevig Mission/1/1918(H1N1) (PDB code: 2WRG; in magenta), H2 HA from A/Singapore/1/1957(H2N2) (PDB code: 2WR7; in orange), H3 HA from A/Aichi/2/68(H3N2) (PDB code: 2YPG; in green), H5 HA from an airborne transmissible mutant of A/Indonesia/5/2005(H5N1) (PDB code: 4K67; in cyan), H7 HA from A/Anhui/1/2013(H7N9) (PDB code: 4BSB; in purple), H9 HA from A/swine/Hong Kong/9/1998(H9N2) (PDB code: 1JSI; in grey), H10 HA from A/Jiangxi/Donghu/346/2013(H10N8) (PDB code: 4QY2; in forest), and H13 HA from A/gull/Maryland/704/1977(H13N6) (PDB code: 4KPS; in pink). The strictly conserved residues among all these HAs within the receptor-binding sites, Y98, W153, H183 and Y195, are shown. Also shown are the main chains of HA_1_ 133 that display different configurations among these structures.

The structures of GD and TW H6 HAs are determined to 2.66 and 2.39 Å, respectively (S1 **Table**). HA is a homotrimer, with each monomer containing two polypeptide chains, HA_1_ and HA_2_ (for clarity, only one monomer is shown in **Fig 1A and 1B**). Both GD and TW H6 HAs have a total of six potential glycosylation sites on each monomer, at HA_1_ 21, 33, 169, 291, 296 and HA_2_ 154. In the structures, we observed five glycans (at HA_1_ 21, 33, 169, 291 and HA_2_ 154) for both H6 HAs (**Fig 1A and 1B**). The glycan at HA_1_ 169 is located at the HA_1_-HA_1_ monomer interface, which was reported to protect H6 HA from trypsin cleavage at R201 [42]. The overall structures of GD and TW H6 HAs are very similar, where the root-mean-square deviation (RMSD) for all Cα atoms is at 0.67 Å (**Fig 1C**).

In agreement with the occurrence of high mutational rates for residues at or surrounding the receptor-binding sites, these regions have the largest structural deviations between GD and TW H6 HAs (**Fig 1C and 1D**). Within the receptor-binding site, GD and TW H6 HAs differ at multiple key locations. These include HA_1_ S133, S136, S137, I155, P186, V187, E190, A193 and G228 for GD H6 HA, whereas R133, T136, N137, V155, L186, D187, V190, N193 and S228 for TW H6 HA (**Fig 1D**). The contributions of these residues to the binding of avian- or human-like receptor analogues will be discussed in the following sections.

Comparison of the receptor-binding sites of different A/HA subtypes of influenza virus reveals that H6 (both GD and TW), H1 and H5 HAs have a bulge in the 130-loop with the main-chain carboxyl group of HA_1_ 133 pointing into the receptor-binding site (**Fig 1E**), likely as a result of the one-residue insertion between HA_1_ 130 and 131 relative to H3 HA (**S3 Fig**). In contrast, other HA subtypes including H2, H3, H7, H9, H10 and H13 have a shorter 130-loop with the main-chain carboxyl group of HA_1_ 133 pointing away from the receptor-binding site (**Fig 1E**). The more hydrophilic environment in this region in the receptor-binding sites of H1, H5 and H6 HAs may help orient the Sia-1 moiety of bound receptors.

### Structures of H6 HAs in complex with avian receptor analogues

**Fig 2:**
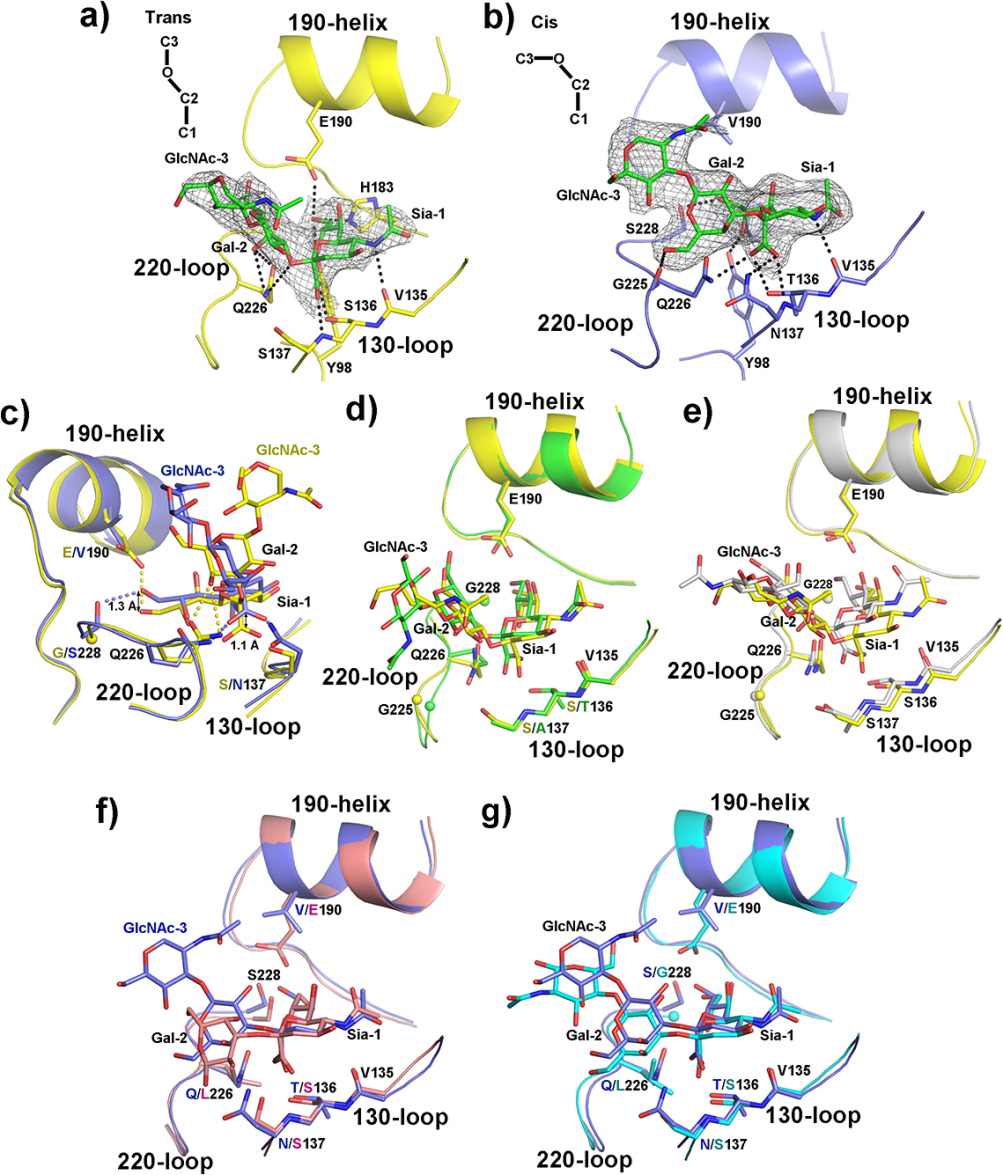
Structural comparison of H6 HAs in binding to avian receptor analogues. **a).** GD H6 HA-LSTa complex. GD H6 HA structure is shown in yellow and LSTa is in green. The composite omit 2*F*_o_-*F*_c_ electron density map for the receptor is shown at 1σ. The hydrogen bonds detected by LIGPLOT are shown as black dashed lines. **b).** TW H6 HA-LSTa complex. TW H6 HA structure is shown in blue and LSTa is in green. The composite omit 2*F*_o_-*F*_c_ electron density map for the receptor is shown at 1σ. The hydrogen bonds detected by LIGPLOT are shown as black dashed lines. **c).** Comparison of LSTa interaction with GD (in yellow) and TW (in blue) H6 HAs. The hydrogen bonds different between these two complexes are shown as dashed lines (yellow dashed lines for hydrogen bonds unique to GD H6 HA-LSTa; blue dashed lines for hydrogen bonds unique to TW H6 HA-LSTa). Highlighted by arrows are the different sitting positions of the LSTa Sia-1 moiety in the receptor-binding sites of GD and TW H6 HAs. **d).** Comparison of avian receptor analogues in GD H6 HA (in yellow) with avian H1 HA from A/WDK/JX/12416/2005(H1N1) (PDB code: 3HTP; in green). **e).** Comparison of avian receptor analogues in GD H6 HA (in yellow) with avian H5 HA from A/Indonesia/5/2005(H5N1) (PDB code: 4K63; in grey). **f).** Comparison of avian receptor analogues in TW H6 HA (in blue) and H5 HA from an airborne transmissible mutant of A/Indonesia/5/2005(H5N1) (PDB codes: 4K66; in red). **g).** Comparison of avian receptor analogues in TW H6 HA (in blue) and H5 HA from a transmissible mutant of A/Vietnam/1203/2004(H5N1) (PDB code: 4BH4; in cyan).

In order to investigate the atomic interactions of H6 HAs with avian-like receptors, we used the pentasaccharide, α(2,3)-linked lactoseries tetrasaccharide a (LSTa), for crystallographic study [43]. The Sia-1, Gal-2 and GlcNAc-3 of the LSTa are clearly visualized in both GD and TW H6 HA structures (**Fig 2A and 2B**). Using LIGPLOT [44], a total of nine hydrogen bonds are detected between GD H6 HA and LSTa and seven hydrogen bonds between TW H6 HA and LSTa (**Table 3, Fig 2A and 2B**). The stronger interactions between GD H6 HA and LSTa are consistent with their relatively higher binding affinity compared to TW H6 HA with LSTa (**Table 2**). Most noticeably, the residues in the receptor-binding sites that differ between TW and GD H6 HAs interact differentially with bound receptors. For instance, the side chain of N137 in TW H6 HA makes one strong hydrogen bond with the O1A atom of Sia-1, which is absent between S137 in GD H6 HA and Sia-1 (**Table 3**). In addition, the side-chain hydroxyl group of S228 in TW H6 HA contributes a strong hydrogen bond with the O9 atom of Sia-1 (**Table 3, Fig 2B and 2C**). On the other hand, different from the small hydrophobic residue V190 in TW H6 HA, GD H6 HA has a large hydrophilic residue E190 that interacts with the O9 atom of Sia-1 (**Table 3, Fig 2A and 2C**).

**Table 3:** Hydrogen bonds between H6 HA proteins and bound receptors.

Receptor	Interaction Pair*	LSTa		LSTc	
analogue		GD H6 HA	TW H6 HA	GD H6 HA	TW H6 HA
**Sia-1**	O1A……S137 N	2.4	**/**	3.1	/
	O1A……N137 N	/	3.0	/	2.8
	O1A……N137 ND2	/	2.7	/	2.8
	O1B……S136 OG	2.4	/	2.8	/
	O1B……T136 OG1	/	2.8	/	2.8
	O1B……S137 N	/	/	3.2	/
	O1B……Q226 NE2	/	3.0	2.9	2.8
	N5………V135 O	2.9	2.9	2.8	2.9
	O8………Y98 OH	/	/	/	3.2
	O9………Y98 OH	2.7	/	3.0	/
	O9………H183 NE2	3.2	/	**/**	/
	O9………E190 OE2	2.9	/	2.7	/
	O9………S228 OG	/	3.0	/	3.0
**Gal-2**	O3………Q226 NE2	3.3	/	/	/
	O4………Q226 NE2	3.2	/	/	/
	O4………Q226 OE1	2.8	/	/	/
	O6………G225 O	/	2.9	/	/

*Hydrogen atoms were added to the structures by Molprobity [45]. These structures were then used to calculate the hydrogen bonds by LIGPLOT [44] with default parameters.

Overall, the Sia-1 moiety of LSTa is ~1.3 Å deeper into the receptor-binding site of GD H6 HA than in TW H6 HA (**Fig 2C**). This lower position of LSTa in GD H6 HA is likely facilitated by several factors: (a). the relatively large hydrophilic residue E190 and the small residue G228 at the back of the receptor-binding site and the relatively small S137 at the front (viewed from the 130-loop) (**Fig 2A and 2C**); (b). the interactions between the LSTa Sia-1 moiety and the extremely conserved residues Y98 and H183 that constitute the base of the receptor-binding site (**Table 3**); and (c). the favorable interactions of Q226 with Gal-2 including the hydrophilic glycosidic oxygen (O3) atom of LSTa in the conventional *trans* conformation (**Table 3**). In sharp contrast, in TW H6 HA, the relatively larger N137 at the front and the smaller hydrophobic V190 and larger S228 at the back of the receptor-binding site (comparing to S137, E190, and G228 of GD H6 HA, respectively) are likely responsible for the higher-sitting position of LSTa, where Q226 contributes a hydrogen bond with Sia-1 O1B atom (**Fig 2B and 2C, Table 3**). LSTa in TW H6 HA adopts a *cis* conformation in favor of the interaction between Gal-2 and the main-chain carboxyl group of G225 (**Fig 2B and 2C, Table 3**). The different conformations of LSTa in GD and TW H6 HAs lead to different paths of exiting the receptor-binding site: starting from Gal-2, LSTa in GD H6 HA extends toward the 130-loop and 220-loop; whereas LSTa in TW H6 HA exits from the N-terminus of the 190-helix (**Fig 2C**).

The *trans* conformation of LSTa in GD H6 HA is similar to that of LSTa in complex with an avian H1 HA from A/WDK/JX/12416/2005(H1N1) (PDB code: 3HTP) [46] (**Fig 2D**) and an avian H5 HA from A/Indonesia/5/2005(H5N1) (PDB code: 4K63) [47] (**Fig 2E**). These HAs have the same residues of V135, E190, G225, Q226 and G228. They differ at HA_1_ 136 and 137: S136 and S137 in GD H6 HA and the avian H5 HA from A/Indonesia/5/2005(H5N1) (PDB code: 4K63) [47], while T136 and A137 in the avian H1 HA from A/WDK/JX/12416/2005(H1N1) (PDB code: 3HTP) [46]. However, the side chains of HA_1_ 136 are in a similar conformation in these structures with the hydroxyl group making equivalent interactions, and the main-chain nitrogen atom of HA_1_ 137 in all three structures contributes a hydrogen bond with Sia-1 (**Fig 2D and 2E**). Together, the smaller side chains at HA_1_ 136 and 137 and the common residues at HA_1_ 135, 190, 225, 226 and 228 in these HA proteins provide a similar receptor-binding site to interact with LSTa.

**Fig 3:**
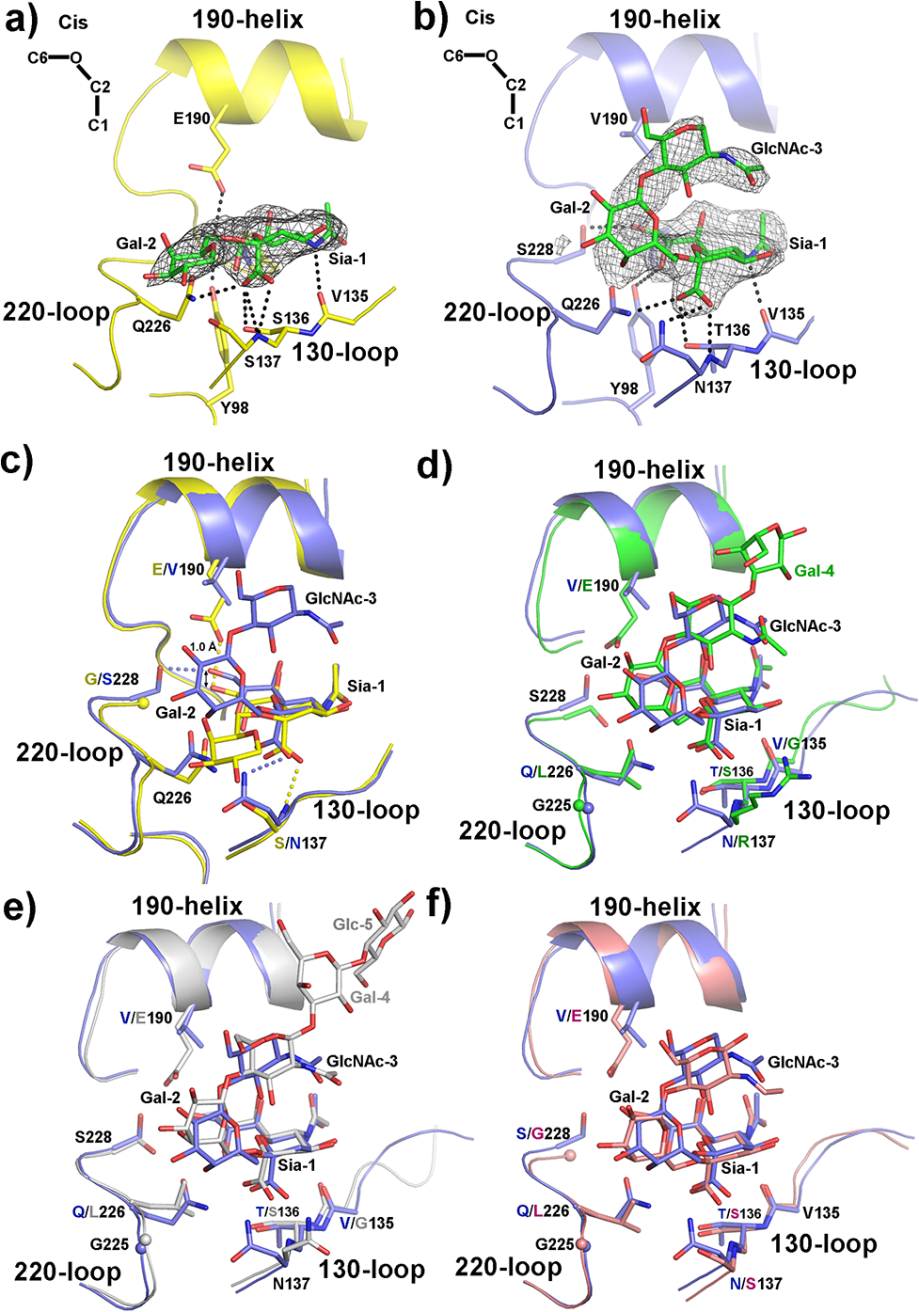
Structural comparison of H6 HAs in binding to human receptor analogues. **a).** GD H6 HA-LSTc complex. GD H6 HA structure is shown in yellow and LSTc is in green. The composite omit 2*F*_o_-*F*_c_ electron density map for the receptor is shown at 1σ. The hydrogen bonds detected by LIGPLOT are shown as black dashed lines. **b).** TW H6 HA-LSTc complex. TW H6 HA structure is shown in blue and LSTc is in green. The composite omit 2*F*_o_-*F*_c_ electron density map for the receptor is shown at 1σ. The hydrogen bonds detected by LIGPLOT are shown as black dashed lines. **c).** Comparison of LSTc interaction with GD (in yellow) and TW (in blue) H6 HAs. The hydrogen bonds different between these two complexes are shown as dashed lines (yellow dashed lines for hydrogen bonds unique to GD H6 HA-LSTc; blue dashed lines for hydrogen bonds unique to TW H6 HA-LSTc). The arrow is to highlight the different sitting positions of the LSTc Sia-1 moiety in the receptor-binding sites of GD and TW H6 HAs. **d).** Comparison of human receptor analogues in TW H6 HA (in blue) and H2 HA from the pandemic A/Singapore/1/1957(H2N2) virus (PDB code: 2WR7; in green). **e).** Comparison of human receptor analogues in TW H6 HA (in blue) and H3 HA from the pandemic A/Aichi/2/68(H3N2) virus (PDB code: 2YPG; in grey). **f).** Comparison of human receptor analogues in TW H6 HA (in blue) and H5 HA from a transmissible mutant of A/Vietnam/1203/2004(H5N1) (PDB code: 4BH3; in red).

On the other hand, the *cis* conformation of LSTa in TW H6 HA closely resembles LSTa in H5 HA from an airborne transmissible mutant of A/Indonesia/5/2005(H5N1) (PDB code: 4K66) [47] (**Fig 2F**) and 3’SLN in H5 HA from a transmissible mutant of A/Vietnam/1203/2004(H5N1) (PDB code: 4BH4) [48] (**Fig 2G**). Both transmissible H5 HAs have V135, S136, S137, E190 and L226, with S228 in one H5 HA (PDB code: 4K66), and G228 in the other H5 HA (PDB code: 4BH4). Conserved interactions among them with avian-like receptors include the hydrogen bonds between Sia-1 and the main-chain carboxyl group of V135, the side-chain hydroxyl group of S136 (both H5 HAs) and T136 (TW H6 HA), and the main-chain nitrogen atom of S137 (both H5 HAs) and N137 (TW H6 HA). Interestingly, the side chain of N137 in TW H6 HA makes a hydrogen bond with Sia-1 O1A atom, while the hydroxyl group of S137 in H5 HA (PDB code: 4K66) forms a hydrogen bond with Gal-2 of LSTa. Of note, the interactions between the side chains of HA_1_ 137 with bound receptors are only observed in a few cases in HA-receptor complexes. Overall, the amino-acid combinations at T136, N137, V190, Q226 and S228 in TW H6 HA and S136, S137, E190, L226 and S/G228 in the transmissible H5 HA mutants have an equivalent effect in recognizing avian-like receptor analogues.

### Structures of H6 HAs in complex with human receptor analogues

We used the pentasaccharide, α(2,6)-linked lactoseries tetrasaccharide c (LSTc), for X-ray crystallographic study of H6 HAs with human-like receptors. In agreement with the weaker binding of GD H6 HA with 6’SLNLN as determined by BLI-based technology (**Table 2**), only the first two sugar rings are clearly visible in the complex structure of GD H6 HA-LSTc (**Fig 3A**). In contrast, TW H6 HA-LSTc has clear density for the first three sugar rings of LSTc (**Fig 3B**).

In the complex structures of H6 HAs with LSTc, there are a total of seven strong hydrogen bonds observed between GD or TW H6 HAs and the receptor that is bound in *cis* conformation (**Fig 3A and 3B, Table 3**). The hydrogen bonds common to both H6 HA-LSTc complexes are with the main-chain carboxyl group of V135, the side-chain hydroxyl group of S136 for GD H6 HA and T136 for TW H6 HA, the side-chain hydroxyl group of Y98, and the side chain of Q226 (**Table 3**). In addition, GD H6 HA has two hydrogen bonds with the main-chain nitrogen atom of S137 and one hydrogen bond with the side chain of E190 (**Fig 3A and 3C, Table 3**), while TW H6 HA has one hydrogen bond with the main-chain nitrogen atom and one with the side-chain amine of N137, and one with the side-chain hydroxyl group of S228 (**Fig 3B and 3C, Table 3**).

As seen in the GD H6 HA-LSTa complex, the back of the Sia-1 moiety of LSTc (the region containing atoms C8, C9 and O9) sits ~1.0 Å lower in GD H6 HA than in TW H6 HA, likely as the result of the different residues at HA_1_ 190 and 228 (E190/G228 in GD H6 HA, and V190/S228 in TW H6 HA) (**Fig 3C**). However, the front of LSTc Sia-1 (atoms C1, O1A and O1B) is at a similar position between GD and TW H6 HAs (**Fig 3C**), which means that LSTc Sia-1 is higher than LSTa Sia-1 for the front in the receptor-binding site of GD H6 HA (**S4A Fig**). Careful inspection of the structures suggests that this is probably due to the different interactions of GD H6 HA Q226 with bound receptors: unlike the strong hydrogen bond between Q226 and the hydrophilic O3 atom of LSTa Gal-2 (**S4B Fig**), the hydrophilic residue Q226 disfavors the hydrophobic C6 atom of LSTc Gal-2 that is placed directly facing Q226 (**S4C Fig**). The resultant higher sitting position of LSTc in the receptor-binding site of GD H6 HA leads to the loss of interactions of Gal-2 with Q226 and Sia-1 with H183 that are observed in the GD H6 HA-LSTa complex and consequently a weaker binding between GD H6 HA and LSTc (**Table 2**). LSTc in GD or TW H6 HA deviates from each other at Gal-2: LSTc Gal-2 in GD H6 HA pointing towards the 130-loop and 220-loop, while LSTc in TW H6 HA exiting the receptor-binding site from the C-terminus of the 190-helix (**Fig 3C**). The *cis* conformation of LSTc in GD H6 HA is significantly different from other HA-LSTc complexes.

The *cis* conformation of LSTc in TW H6 HA is similar to that of LSTc in H2 HA from the pandemic A/Singapore/1/1957(H2N2) virus (PDB code: 2WR7) [49] (**Fig 3D**), LSTc in H3 HA from the pandemic A/Aichi/2/68(H3N2) virus (PDB code: 2YPG) [50] (**Fig 3E**), and 6’SLN in H5 HA from a transmissible mutant of A/Vietnam/1203/2004(H5N1) (PDB code: 4BH3) [48] (**Fig 3F**). They all share similar interactions with HA_1_ Y98 (side-chain hydroxyl group), HA_1_ 135 (main-chain carboxyl group), HA_1_ 136 (side-chain hydroxyl group), and HA_1_ 137 (main-chain nitrogen atom). However, they differ at multiple sites within the receptor-binding site, particularly V190 and Q226 in TW H6 HA and E190 and L226 in all other HAs. Both the H3 HA from the pandemic A/Aichi/2/68(H3N2) virus (PDB code: 2YPG) [50] and the H5 HA from the transmissible mutant of A/Vietnam/1203/2004(H5N1) (PDB code: 4BH3) [48] have hydrogen bonds between E190 and Sia-1. Furthermore, TW H6 HA and H3 HA from the pandemic A/Aichi/2/68(H3N2) virus (PDB code: 2YPG) [50] also have in common a hydrogen bond between S228 and Sia-1. In contrast, the side chain of N137 in TW H6 HA contributes a hydrogen bond with LSTc Sia-1 (**Table 3**).

It is well accepted that the hydrophobic residue at HA_1_ L226 in H2 and H3 HAs favors the binding of human-like α(2,6)-receptors in *cis* conformation with the hydrophobic C6 atom facing the 220-loop, while the hydrophilic Q226 stabilizes the binding of avian-like α(2,3)-receptors in the classical *trans* conformation where the hydrophilic glycosidic oxygen (O3) atom pointing towards the 220-loop [17,20]. In TW H6 HA, the combination of N137, V190 and S228 results in a similarly positioned Sia-1 moiety of LSTc, thus likely alleviating the unfavorable interaction between Q226 and LSTc in *cis* conformation and resulting in substantially enhanced binding to human-like receptors (**Table 2**). It is interesting to note that out of the total of 35 H6 HA sequences isolated from Taiwan in the past 14 years, 33 of them constitute a distinct branch within Group II (**S1 Fig**). Within this Taiwan-specific branch, 26 HA sequences (accounting for 6% of all Group II sequences) harbor the N137, V190 and S228 triad as observed in the human-infecting TW H6 HA.

## Discussion

Both GD and TW H6 viruses exhibit a potential threat to humans: GD H6 virus, despite its avian origin, was able to transmit in guinea pigs through direct inoculation or contact [13], while TW H6 virus caused the first human infection in 2013 [4–6]. In this study, we aimed to evaluate their true potential for human infections and the molecular mechanisms that H6 HAs may harness in their natural evolution to allow airborne transmission among humans.

Using BLI technology, we measured the binding affinity of GD and TW H6 HA proteins with avian-like 3’SLNLN and human-like 6’SLNLN receptor analogues (**Table 2**). GD H6 HA is primarily an avian HA with a high binding affinity for 3’SLNLN (*K*_D_ of 0.097 μM). Its binding affinity of *K*_D_ of 5 μM for 6’SLNLN, albeit a bit lower, is still sufficient to support the transmission among guinea pigs through direct inoculation or contact [13]. On the other hand, comparing to GD H6 HA, TW H6 HA has a substantially enhanced binding affinity for 6’SLNLN (*K*_D_ of 0.27 μM) and slightly weakened binding for 3’SLNLN (*K*_D_ of 0.4 μM) (**Table 2**). Although TW H6 HA is still an avian HA with considerable binding for avian-like receptors, its slight preference for human-like receptors suggests that it may be on the path towards a full adaptation in humans, thus posing a substantial threat to human health.

The two H6 HA proteins have the following key residues in the receptor-binding sites: **V135,** S136, S137, E190, **G225, Q226** and G228 for GD H6 HA, and **V135,** T136, N137, V190, **G225, Q226** and S228 for TW H6 HA (the common residues are highlighted in boldface). Their complex structures with avian-like LSTa receptor and human-like LSTc receptor revealed that among the residues that differ between GD and TW H6 HAs, while HA_1_136 interacts with bound receptors via main-chain atoms, the side-chain atoms of HA_1_ 137, 190, and 228 are the major discriminating factors in binding to avian- or human-like receptors (**Figs 2 and 3, Table 3**). For instance, the side-chain carboxyl group of E190 forms a strong hydrogen bond with the Sia-1 O9 atom of both LSTa and LSTc of GD H6 HA. On the other hand, in the complex of TW H6 HA with LSTa or LSTc, N137 contributes two strong hydrogen bonds with the Sia-1 moiety, one by the main-chain nitrogen atom and the other by the side-chain amine group (**Figs 2 and 3, Table 3**). Furthermore, the side-chain hydroxyl group of S228 of TW H6 HA forms a strong hydrogen bond with the Sia-1 O9 atom of both LSTa and LSTc.

In the GD H6 HA-LSTa complex (**Fig 2A and 2C**), the combination of S137, E190 and G228 results in a much deeper location of LSTa inside the receptor-binding site, thus allowing additional interactions with Y98, H183 and Q226. In this conformation, the hydrophilic residue Q226 helps stabilize the hydrophilic glycosidic oxygen (O3) atom of LSTa in *trans* conformation (**Table 3**). In marked contrast, in the GD H6 HA-LSTc complex (**Fig 3A and 3C**), the hydrophilic residue Q226 repels the hydrophobic C6 atom of bound LSTc in its conventional *cis* conformation, leading to a higher-sitting position for the front of Sia-1 (including atoms C1, O1A and O1B) in the receptor-binding site (**S4A Fig**), thus losing some of the strong interactions with Y98, H183 and Q226, and resulting in a weakened binding for human-like receptors (**Table 2**). Nevertheless, the amino-acid composition of HA_1_ S137, E190, and G228 in the receptor-binding site of GD H6 HA allows the binding to both human- and avian-like receptors, albeit with a slight preference for avian-like receptors.

Different from GD H6 HA, the combination of N137, V190 and S228 in TW H6 HA leads to a higher-sitting position of LSTa and LSTc within the receptor-binding site. This higher-sitting position alleviates the discriminating effect of HA_1_ 226 for avian or human-like receptors. Thus LSTa and LSTc bind similarly in the receptor-binding site of TW H6 HA. This explains why TW H6 HA, with the hydrophilic residue Q226, interacts with human-like receptors in a similar way as pandemic H2 and H3 and transmissible H5 HAs that possess the hydrophobic residue L226.

While this manuscript was in preparation or under review, three studies on structural and receptor binding of H6 HAs have been published [51–53]. One study reported the complex structures of an avian H6 HA in complex with 3’SLN and LSTa (the first three sugars were visible), and TW H6 HA with 6’SLN (where the first Sia-1 was observed) [52]. Another study reported the complex structures of TW H6 HA in complex with 3’SLN and LSTa (in which the first three sugar rings were visualized) and with 6’SLN (in which both Sia-1 and Gal-2 were visible) [53]. Both studies did not detect the binding of TW H6 HA with human-like α(2,6)-receptors, likely due to the relatively low HA concentration that was used in the experiments [52,53]. A third study compared the structures of an avian H6 HA (A/chicken/Taiwan/A2837/2013(H6N1)) and TW H6 HA with 3’SLNLN (where the first three and four sugar rings were visible, respectively), or with 6’SLNLN (in which the first two sugar rings were seen) [51]. This study found that TW H6 HA has a slight preference for human-like receptors [51], which agrees with our BLI-based measurement of binding affinity (**Table 2**). The employment of a wide range of protein concentrations used therein [51] and in our study is the key for obtaining reliable measurement of the intrinsically weak binding affinity between HA and its receptors. The discrepancy in the absolute *K*_D_ values between Wang *et al*. [51] and our study is likely the results of using different types of instruments and probably other experimental conditions.

In our study, the HA-receptor complex structures have enabled the visualization of the first three sugar rings of bound avian-like LSTa in both GD and TW H6 HAs, and the first two and three sugar rings of bound human-like LSTc in GD and TW H6 HAs, respectively. The amino-acid composition of HA_1_ S137, E190, and G228 in the receptor-binding site of GD H6 HA allows the binding to both human- and avian-like receptors, albeit with a slight preference for avian-like receptors. The presence of this amino-acid composition in Group I and the majority of Group II H6 HA sequences helps explain the broader host range of H6 viruses than other subtypes of influenza A virus [7]. In addition, the combination of residues at HA_1_ N137, V190 and S228 of TW H6 HA may alleviate the effect of HA_1_ 226 of H2 and H3 HAs in discriminating human- or avian-like receptors. Consequently, different from GD H6 HA, TW H6 HA has a slight preference for human-like receptors, thus may represent an evolutionary intermediate where a minimal set of mutations may endorse a complete human adaptation. In particular, out of the total of 35 non-redundant H6 HA sequences detected in Taiwan during 2000~2014, 26 of them (at~74%) carry the N137/V190/S228 triad, thus suggesting an elevated threat of this subgroup of H6 viruses to human health.

It is important to note that all current studies on HA are limited to a small subset of milk sugars that are available in good quantity. Given the considerable variations in carbohydrate composition and modifications of natural receptors that influenza HA recognizes [54], it is our hope that such studies can be expanded to a larger variety of receptors in the future to allow a more detailed structural and functional characterization of HA-receptor interactions and more accurate prediction of epidemic and pandemic potential of influenza virus field isolates. The overall strong binding of H6 HAs to both avian- and human-like receptors tested in this study and the preference for human-like receptors of H6 viruses isolated from Taiwan justify the need for diligent surveillance of H6 viruses around the world with particular attention to Taiwan isolates.

## Materials and Methods

### Sequence analysis of H6 HAs

The complete H6 HA amino-acid sequences from January 1, 2000 to July 14, 2014 were retrieved from GISAID. The HA ectodomains were aligned by Clustal Omega [55]. Jalview [56] was used to remove redundant sequences and generate the phylogenetic tree for the 737 non-redundant H6 HA sequences.

### Expression and purification of GD and TW H6 HA proteins

The genes encoding the ectodomains of A/Taiwan/2/2013(H6N1) (TW H6) and A/chicken/Guangdong/S1311/2010(H6N6) (GD H6) HA proteins were assembled [57] from oligos ordered from Integrated DNA Technologies. The H6 HA genes were inserted into pFastBac1 vector that was modified to contain a C-terminal T4 fibritin (foldon) and His_6_-tag [58,59]. The recombinant baculoviruses were made according to the manufacturer’s instruction (Invitrogen). High Five cells were infected at a multiplicity of infection of ~1.0 for 40 hours and the culture was harvested. The supernatant was dialyzed against 20 mM Tris, pH 7.5, 50 mM NaCl followed by incubating with Ni-NTA resin (Thermo Scientific). The HA-bound resin was washed with wash buffer (20 mM Tris, pH 7.5, 50 mM NaCl and 15 mM imidazole) and digested by trypsin (at a weight ratio of about 1:1000) at room temperature overnight to remove the C-terminal foldon and His_6_-tag, which also cleaved HA into HA_1_ and HA_2_. The cleaved HA was further purified by Mono Q 4.6/100 PE and Superdex 200 10/300 GL (GE Healthcare).

### Crystallization and structure determination of H6 HA proteins

The H6 HA trimer peak from gel filtration was concentrated to about 10 mg/mL and buffer exchanged to 0.1 M Bis-Tris propane, pH 8.0. The GD H6 HA crystal was grown by mixing the same volume of 0.1 M Bis-Tris propane, pH 8.0 and 2.3 M (NH_4_)_2_SO_4_ in hanging drops. The solution for growing TW H6 HA crystal was 0.1 M Bicine, pH 9.0, 10% PEG 20000 and 2% 1,4-Dioxane. The crystals were soaked with 5 mM LSTa or LSTc (Carbosynth) for 2 hours and flash frozen in the crystallization solution supplemented with 25% glycerol (GD H6 HA) or 7.5% glycerol (TW H6 HA). The diffraction data were collected at LS-CAT in Advanced Photon Source. The reflections were integrated by using Mosflm [60]. The CCP4 programs [61] were used to scale and truncate the reflections. The H1 HA structure (PDB accession code: 1RUZ) [34] was used as the search model for molecular replacement using PHENIX [62]. The model was refined and manually built by using PHENIX [62] and COOT [63], respectively.

### Receptor binding affinity of H6 HAs

The OCTET streptavidin sensors (Pall ForteBio) were loaded with biotinylated 3’-SLNLN or 6’-SLNLN (Consortium of Functional Glycomics, cfg_rRequest_2413) followed by association with H6 HA in kinetic buffer (PBS with 0.01% bovine serum albumin) for 10 minutes and dissociation in the kinetic buffer (without HA) for 10 minutes. Different HA concentrations (0.225~18 μM) were used to obtain a full range of association and dissociation curves. The association rate (*K*_on_) and dissociation rate (*K*_off_) were obtained by fitting the association and dissociation curves using the system software and a 1:1 binding model. The equilibrium dissociation constants *K*_D_ were calculated from the ratio of *K*_off_ and *K*_on._

## Supporting Information

S1 Fig Phylogenic tree of 737 non-redundant HA sequences from H6 viruses isolated between 2000~2014. (TIF)

S2 Fig Representative binding curves of GD (a) and TW (b) H6 HA proteins with 3’SLNLN and 6’SLNLN receptor analogues. The corresponding HA concentrations are: 0.225 μM GD H6 HA with 3’SLNLN (blue curve in **a)**); 9 μM GD H6 HA with 6’SLNLN (red curve in **a)**); 9 μM TW H6 HA with 3’SLNLN (blue curve in **b)**) or 6’SLNLN (red curve in **b)**)
(TIF)

S3 Fig Structure-based sequence alignment of different A/HA subtypes. * indicates an insertion between HA_1_ 130 and 131 in H1, H5 and H6 HAs.
(TIF)

S4 Fig Structural comparison of GD H6 HA in binding to receptor analogues. **a).** Comparison of the Sia-1 moiety of LSTa (in yellow) and LSTc (in grey) in GD H6 HA. Arrows highlight the different sitting positions of Sia-1. **b).** GD H6 HA-LSTa complex to highlight the hydrogen bond between Q226 and the O3 atom of LSTa Gal-2 (as yellow dashed line). **c).** GD H6 HA-LSTc complex to highlight the distance between Q226 and the hydrophobic C6 atom of LSTc Gal-2 (by a double-headed arrow).
(TIF)

S1 Table Statistics of data collection and refinement of GD and TW H6 HA structures (DOCX)
