# Supplementary figures and images for: Structural and Functional Studies of Influenza Virus A/H6 Hemagglutinin
Source: PLoS One. 2015 Jul 30;10(7):e0134576. doi: 10.1371/journal.pone.0134576 (PMC4520562; doi:10.1371/journal.pone.0134576)

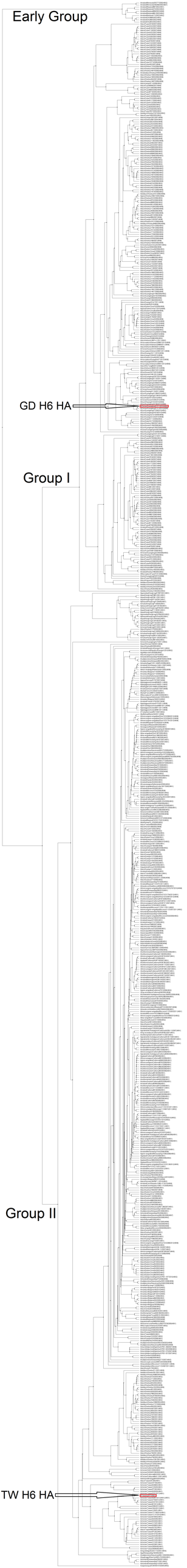

Supplement: S1 Fig — (TIF) [file pone.0134576.s001.tif]

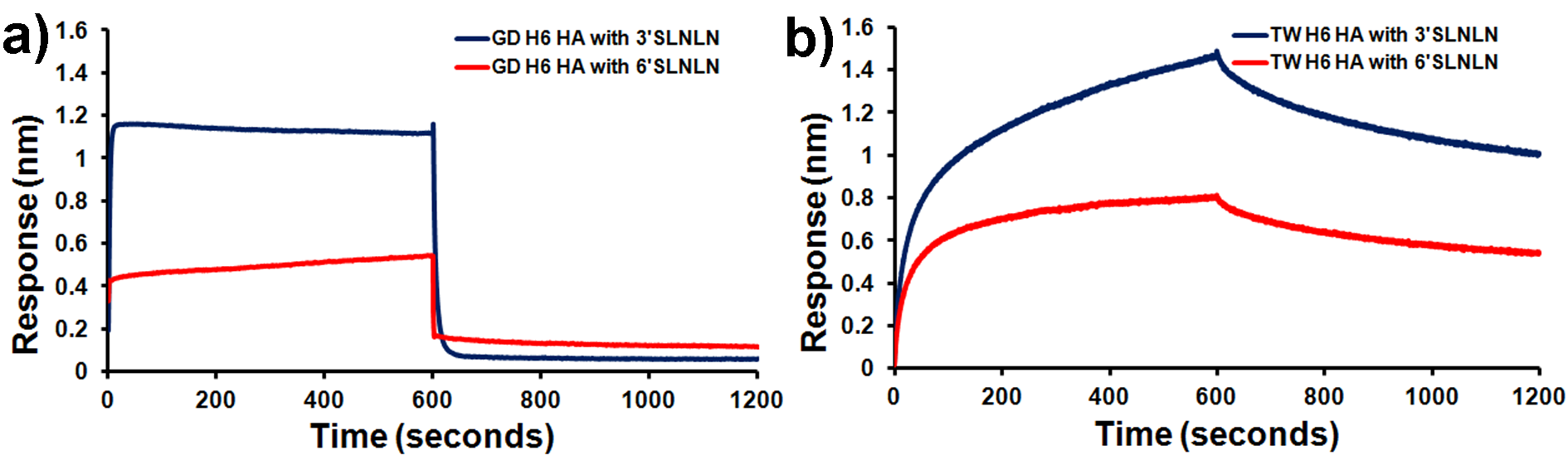

Supplement: S2 Fig — The corresponding HA concentrations are: 0.225 μM GD H6 HA with 3’SLNLN (blue curve in a)); 9 μM GD H6 HA with 6’SLNLN (red curve in a)); 9 μM TW H6 HA with 3’SLNLN (blue curve in b)) or 6’SLNLN (red curve in b)) (TIF) [file pone.0134576.s002.tif]

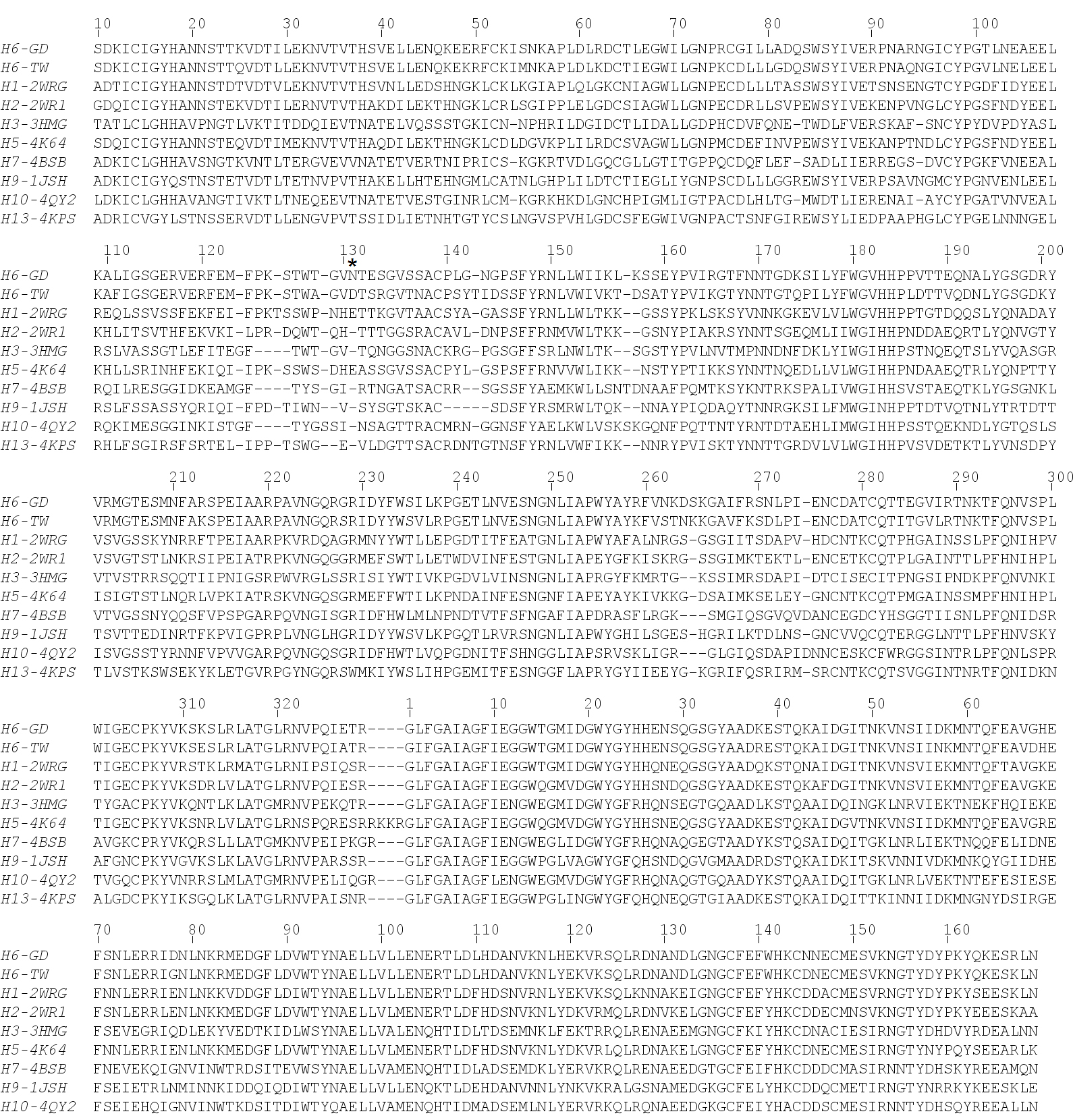

Supplement: S3 Fig — * indicates an insertion between HA1 130 and 131 in H1, H5 and H6 HAs. (TIF) [file pone.0134576.s003.tif]

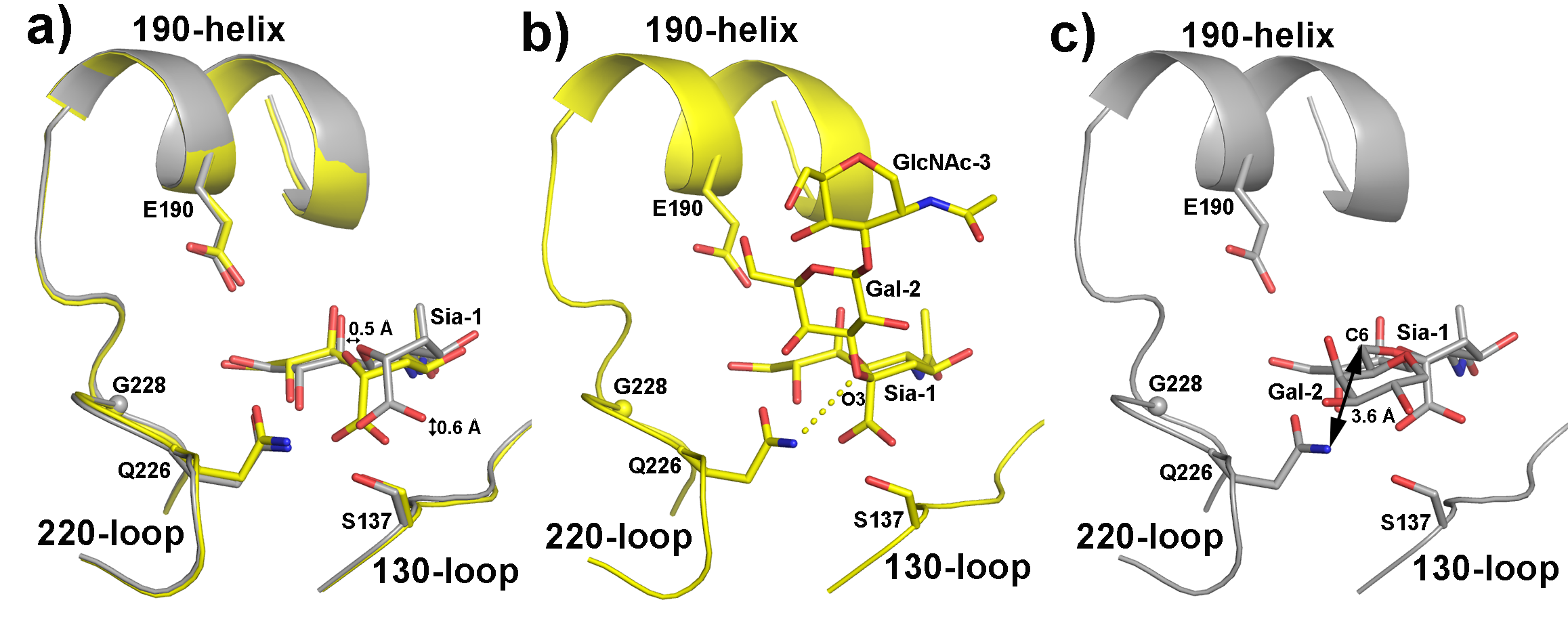

Supplement: S4 Fig — a). Comparison of the Sia-1 moiety of LSTa (in yellow) and LSTc (in grey) in GD H6 HA. Arrows highlight the different sitting positions of Sia-1. b). GD H6 HA-LSTa complex to highlight the hydrogen bond between Q226 and the O3 atom of LSTa Gal-2 (as yellow dashed line). c). GD H6 HA-LSTc complex to highlight the distance between Q226 and the hydrophobic C6 atom of LSTc Gal-2 (by a double-headed arrow). (TIF) [file pone.0134576.s004.tif]
