# Supplementary material for: Structural and Functional Studies of Influenza Virus A/H6 Hemagglutinin
Source: PLoS One. 2015 Jul 30;10(7):e0134576. doi: 10.1371/journal.pone.0134576 (PMC4520562; doi:10.1371/journal.pone.0134576)
Supplement: S1 Table — (DOCX) [file pone.0134576.s005.docx]

S1 Table. Statistics of data collection and refinement of GD and TW H6 HA structures

|  | GD H6 HA | GD H6 HA-LSTa | GD H6 HA-LSTc | TW H6 HA | TW H6 HA-LSTa | TW H6 HA-LSTc |
| --- | --- | --- | --- | --- | --- | --- |
| Wavelength (Å) | 0.97849 | 0.97850 | 0.97849 | 0.97856 | 0.97872 | 0.97872 |
| Resolution range (Å) | 49.69-2.66 (2.80-2.66) | 59.13-2.78 (2.93-2.78) | 47.95-2.89 (3.05-2.89) | 63.25-2.39 (2.52-2.39) | 42.26-2.55 (2.69-2.55) | 37.69-2.43 (2.56-2.43) |
| Space group | *P*2_1_2_1_2_1_ | *P*2_1_2_1_2_1_ | *P*2_1_2_1_2_1_ | *P*3 | *P*3 | *P*3 |
| Unit cell a, b, c (Å)  α , β , γ (°) | 69.3, 137.4, 198.9  90, 90, 90 | 69.4, 137.8, 199.1  90, 90, 90 | 69.6, 138.6, 199.1  90, 90, 90 | 114.0, 114.0, 164.4  90, 90, 120 | 114.0, 114.0, 163.2  90, 90, 120 | 113.7, 113.7, 163.2  90, 90, 120 |
| Total reflections | 237,232 (31,611) | 199,895 (29,090) | 144,835 (21,076) | 332,533 (42,484) | 276,773 (40,289) | 318,631 (46,323) |
| Unique reflections | 53,602 (7,114) | 48,630 (7,035) | 39,833 (5,835) | 94,738 (13,854) | 77,363 (11,317) | 88,853 (12,981) |
| Multiplicity | 4.4 (4.4) | 4.1 (4.1) | 3.6 (3.6) | 3.5 (3.1) | 3.6 (3.6) | 3.6 (3.6) |
| Completeness (%) | 96.9 (89.7) | 99.7 (100.0) | 91.2 (92.9) | 100.0 (100.0) | 100.0 (100.0) | 100.0 (100.0) |
| Mean I/σI | 12.4 (2.7) | 11.3 (2.4) | 8.3 (1.7) | 9.0 (2.0) | 8.6 (2.2) | 9.7 (2.5) |
| Wilson *B*-factor (Å^2^) | 40.5 | 47.2 | 50.8 | 30.6 | 32.8 | 31.2 |
| *R*_sym_ | 0.104 (0.487) | 0.119 (0.526) | 0.151 (0.653) | 0.134 (0.507) | 0.132 (0.492) | 0.124 (0.499) |
| *R*_work_ | 0.202 | 0.196 | 0.225 | 0.183 | 0.201 | 0.200 |
| *R*_free_ | 0.245 | 0.251 | 0.274 | 0.210 | 0.228 | 0.222 |
| Number of atoms | 12,460 | 12,368 | 11,950 | 8,975 | 8,841 | 9,037 |
| Macromolecules | 11,870 | 11,857 | 11,752 | 7,916 | 7,916 | 7,916 |
| Ligands | 266 | 327 | 198 | 254 | 355 | 344 |
| Water | 324 | 184 | N/A | 805 | 570 | 777 |
| Protein residues | 1,492 | 1,490 | 1,481 | 994 | 994 | 994 |
| RMSD (bonds) (Å) | 0.004 | 0.005 | 0.005 | 0.004 | 0.004 | 0.004 |
| RMSD (angles) (°) | 0.845 | 0.801 | 1.006 | 0.843 | 0.776 | 0.746 |
| Ramachandran favored (%) | 98.58 | 97.29 | 96.53 | 98.17 | 98.07 | 97.36 |
| Ramachandran outliers (%) | 0 | 0 | 0 | 0 | 0 | 0 |
| Clashscore | 4.57 | 4.01 | 5.79 | 3.41 | 6.36 | 4.36 |
| Average *B*-factor (Å^2^) | 41.0 | 46.9 | 43.2 | 38.0 | 39.8 | 37.7 |
| Macromolecules | 40.0 | 45.7 | 42.9 | 36.4 | 38.5 | 36.4 |
| Solvent | 38.1 | 41.3 | N/A | 43.2 | 43.5 | 42.6 |
| PDB accession code | 5BNY | 5BQY | 5BQZ | 5BR0 | 5BR3 | 5BR6 |

Statistics for the highest-resolution shell are shown in parentheses. N/A means “not applicable”.
